# Antithrombotic Therapy in Transcatheter Aortic Valve Replacement

**DOI:** 10.3389/fcvm.2019.00073

**Published:** 2019-05-31

**Authors:** Roberto Valvo, Giuliano Costa, Corrado Tamburino, Marco Barbanti

**Affiliations:** Division of Cardiology, Policlinico–Vittorio Emanuele Hospital, University of Catania, Catania, Italy

**Keywords:** TAVR, bleeding, stroke, antiplatelet therapy, anticoagulant therapy

## Abstract

Transcatheter aortic valve replacement (TAVR) has recently emerged as an effective alternative to medical treatment or surgical aortic valve replacement in all symptomatic patients with severe aortic stenosis and high or prohibitive risk and in intermediate risk when transfemoral access is feasible. Patients undergoing TAVR are often at high risk for either bleeding or cerebrovascular complications, or both, so adjuvant antithrombotic therapies are commonly used before, during and after the procedure. Today, there is no clear evidence on the best antithrombotic regimen in this context. In this review, we will try to go through the mechanisms involved in bleeding and embolic complications and we will discuss the current points of antithrombotic treatment in patients during and after TAVR, with or without oral anticoagulation (OAC) indication.

## Introduction

**Figure 1:**
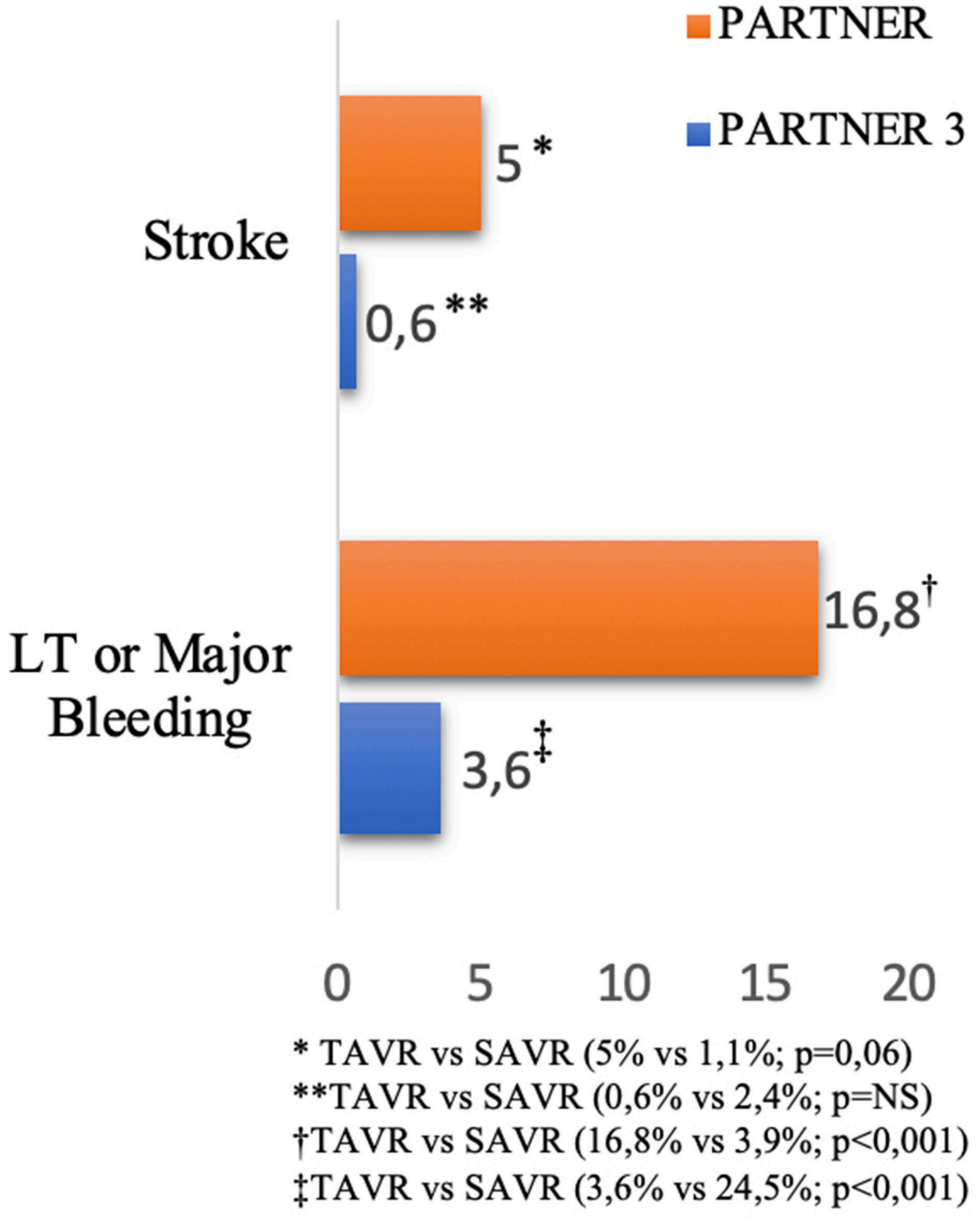
Incidence of stroke and bleeding after TAVR at 30 days reported in PARTNER and PARTNER 3 Trials (2, 4).

Transcatheter aortic valve replacement (TAVR) has emerged as an effective alternative to medical treatment or surgical aortic valve replacement (SAVR) in elderly symptomatic patients with severe aortic stenosis when transfemoral access is available (1–3). The first randomized PARTNER trial showed that TAVR offers better survival rates than medical therapy, but it was associated with a high incidence of stroke (5% at 30 days) and bleeding (16.8% at 30 days) in high surgical risk patients (4). More recently, the PARTNER 3 trial showed a lower incidence of stroke (0.6% at 30 days) and life-threatening or major bleeding (3.6% at 30 days), probably due to the low risk patients and to the new generation devices (Figure 1) (2). Nevertheless, bleeding and ischemic complications remain significant after TAVR, which are related to increased morbidity and mortality (5). Adjuvant antithrombotic therapies are commonly used during and after TAVR, with the aim to decrease the risk of thromboembolic cerebrovascular events and valve thrombosis, but consequently increasing the risk of bleeding. Nevertheless, the optimal anti-thrombotic regimen during and after TAVR remains a matter of debate. The European Society of Cardiology (ESC) (6) guidelines recommend dual antiplatelet therapy (DAPT) for 3–6 months followed by single antiplatelet therapy (SAPT) lifelong after TAVR in patients who are not candidates to oral anticoagulation (Class of recommendations IIa—Level of evidence C), and only SAPT in high bleeding risk patients (Class of recommendations IIb—Level of evidence C). In patients with indication for oral anticoagulation (OAC), such therapy is recommended lifelong (Class of recommendations I—Level of evidence C) (Table 1) (1). In the US, the guidelines from AHA/ACC recommend anticoagulation with a VKA to achieve an international normalized ratio of 2.5 in patients at low risk of bleeding for at least 3 months (Class of recommendations IIb—Level of evidence B) or DAPT for 6 months followed by SAPT lifelong (Class of recommendations IIb—Level of evidence C). Currently, antiplatelet therapy with aspirin and clopidogrel is the most adopted antithrombotic regimen for patients undergoing TAVR with no indication for OAC (7). However, the current guidelines are largely based upon empirical information rather than evidence-based data. Further, the increasing use of most recent P2Y12 inhibitors and new oral anticoagulants (NOACs) in clinical practice will introduce variability in treatment. The randomized trials are the best path forward to determine the balance between the risks and the efficacy of antithrombotic and/or anticoagulant treatment in this population.

**Table 1:** Antithrombotic treatment recommendations after TAVR.

**Document**	**Year**	**Recommendations**				**Duration**
ESC/EACTS guidelines	2017	No OAC	DAPT should be considered, followed by SAPT	IIa	C	3–6 months DAPT, then SAPT lifelong
			SAPT may be considered after TAVR in the case of high bleeding risk	IIb	C	No specific recommendation
		OAC	Oral anticoagulation is recommended	I	C	Lifelong
ACC/AHA guidelines	2017	No OAC	Clopidogrel 75 mg daily may be reasonable in addition to aspirin 75-100 mg daily	IIb	C	6 month DAPT, then aspirin lifelong
			Anticoagulation with VKA (INR 2.5) may be reasonable in patients at low risk of bleeding	IIb	B	At least 3 months
		OAC	No specific recommendations			No specific recommendations

*DAPT, dual antiplatelet therapy; SAPT, single antiplatelet therapy; OAC, oral anticoagulant; Green, Class of recommendations I; Yellow, Class of recommendations IIa; Orange, Class of recommendations IIb; Blue, Level of evidence B; Light Blue, Level of evidence C*.

### Risk of Bleeding and Cerebrovascular Events

The rates of major and life-threatening bleeding, based on Valve Academic Research Consortium (VARC) definitions (Supplementary Table 1) (8), in the peri-procedural period have been reported as high as 15–32 and 5–16%, respectively (9). The mechanisms of peri and post procedural bleeding complications in TAVR seems to correlate mainly to vascular access site complications, related to the use of high-profile delivery systems in a very elderly population. This aspect suggests that meticulous planning of the procedure in terms of evaluation of vascular access site by multidetector computed tomography (MDCT) is crucial to identify the most appropriate puncture site to reduce the risk of bleeding events and vascular complications. Nevertheless, a non-negligible proportion of patients receive post-procedural blood transfusion despite having no evident source of bleeding directly related with the vascular access site (typically small gastrointestinal or/and genitourinary blood loss) (10). A sub-analysis of the PARTNER trial showed that after 30 days, major late bleeding complications (MLBCs) occurred in 5.9% of TAVR patients at a median time of 132 days (interquartile range: 71–230 days) after the index procedure; the most frequent type of MLBCs are gastrointestinal complications and neurological complication (5).

On the other hand, stroke is an important adverse event associated with TAVR. One of the pathophysiological mechanisms underlying cerebrovascular events is that the native stenotic aortic valve has a large amount of tissue factor and thrombin that contribute to the thrombogenicity. Unlike SAVR, the native valve remains *in situ* after TAVR and its manipulation during the implantation of the new valve predisposes to greater exposure and/or embolization of its component in the peripheral circulation. Furthermore, the interaction between the valve prosthesis and the native aortic valve may generate flow turbulence that predisposes to thrombus development, especially when there is a valve-patient mismatch (11). Moreover, the thrombophilic state induced by the devices used in TAVR may also stimulate thrombus formation through platelet aggregation and subsequent activation of the coagulation pathway (12, 13). Finally, it is important to recognize that many patients who have aortic stenosis may also have other causes for an ischemic stroke such as hypertension, diabetes, age, or other conditions, including atrial fibrillation, which is a potent risk factor for cardio-embolic stroke (14).

## Current Antithrombotic Management During TAVR

For the elevated risk of thromboembolic events, anticoagulation is required during TAVR. In daily practice, unfractionated heparin (UFH) has been used as the standard procedural anticoagulation regimen for TAVR. Usually, anticoagulation therapy starts after insertion of the regular sheaths and prior to placement of the large sheath into the vessel, and is continued to maintain an activated clotting time (ACT) of >300 s, recommended by the American College of Cardiology Foundation/American Association for Thoracic Surgery/Society for Cardiovascular Angiography and Interventions/Society of Thoracic Surgeons (ACCF/AATS/SCAI/STS) expert consensus document on TAVR (14). It must be said that practice patterns vary, being guidelines based on expert consensus rather than on evidence from RCTs. The ACCF/AATS/SCAI/STS expert consensus document recommends heparin anticoagulation to be reversed after the procedure by administration of protamine sulfate at a milligram-to-milligram neutralization dose.

Direct thrombin inhibition with bivalirudin was studied in alternative to heparin as the procedural anticoagulant agent in this setting. However, the BRAVO-3 (Bivalirudin vs. Heparin Anticoagulation in Transcatheter Aortic Valve Replacement) demonstrated that UFH should remain the standard of care in patients undergoing TAVR as bivalirudin did not reduce rates of major bleeding at 48 h or adverse cardiovascular events within 30 days (15). Furthermore, although bivalirudin may be useful in the high bleeding risk patients undergoing TAVR, bleeding and life-threatening vascular complications occurring during TAVR, such as peripheral vascular rupture, annulus rupture, or cardiac tamponade, often require rapid reversal of anticoagulation, which is impossible with bivalirudin, despite the short half-life of this drug. For this reason, bivalirudin has to be considered as alternative anticoagulant only for patients not able to receive heparin. Anyway, the expansion of TAVR procedures worldwide necessitates dedicated clinical investigation in the field of peri-procedural anticoagulant treatment, with the goal of building appropriate practice guidelines and further improving clinical outcomes.

## Current Antithrombotic Management After TAVR

**Figure 2:**
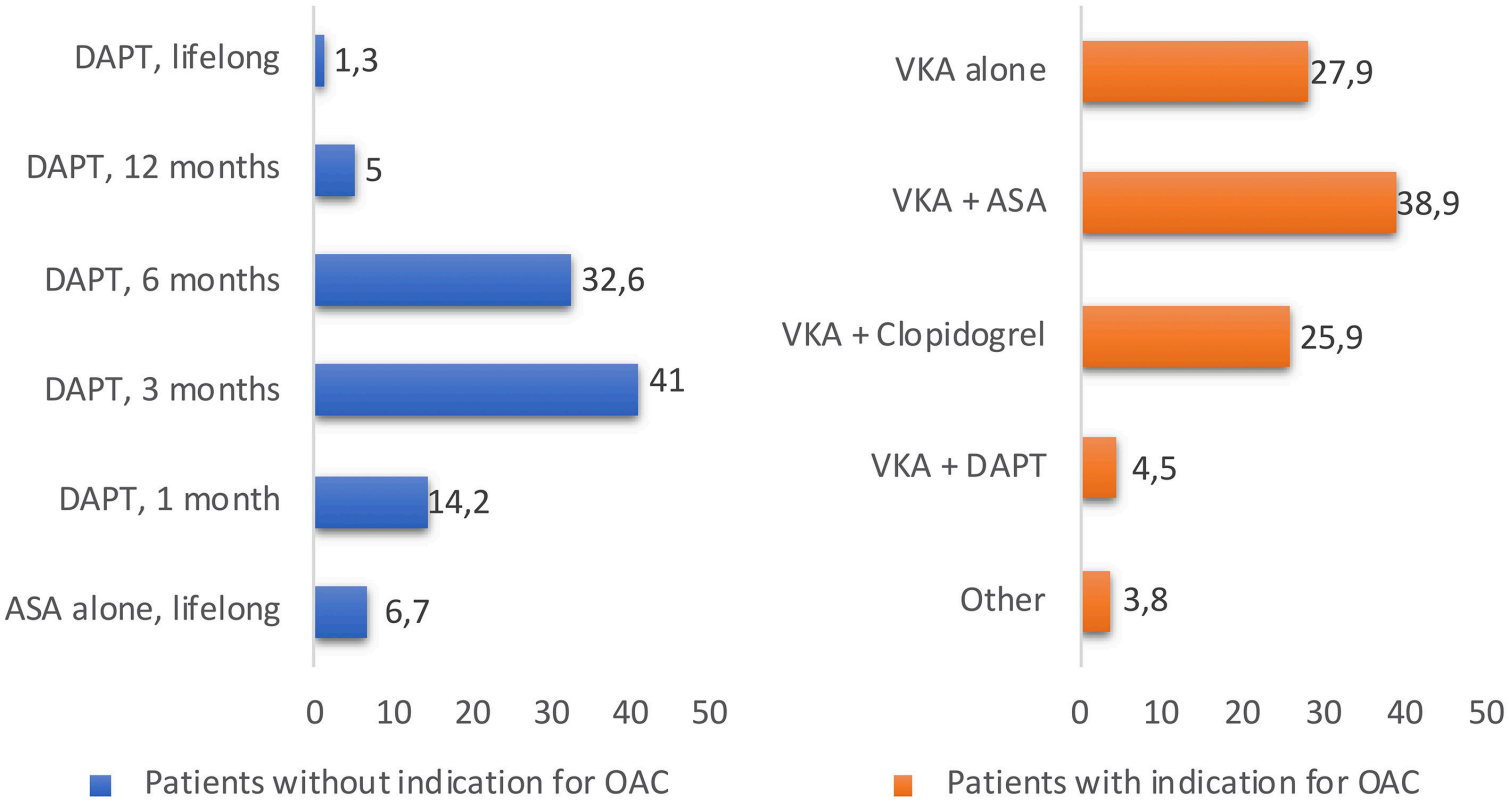
The duration of DAPT or OAC therapy varied widely among centers, reported in the study “Evaluation of current practices in transcatheter aortic valve implantation: The WRITTEN (19).” DAPT, dual antiplatelet therapy; ASA, acetylsalicylic acid; OAC, oral anticoagulant; VKA, Vitamin K antagonists.

**Figure 3:**
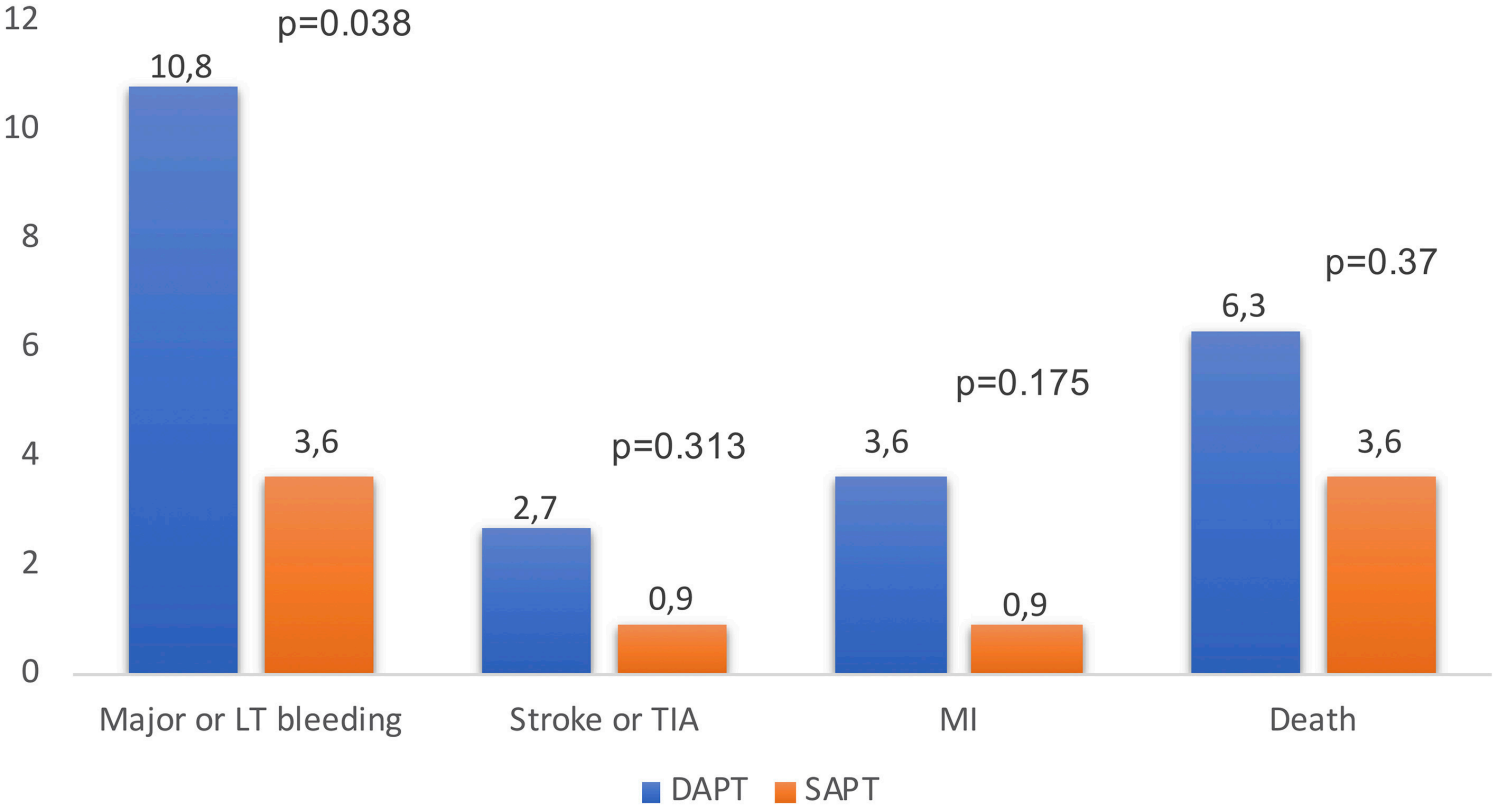
Rates of major or life-threatening events, ischemic stroke or TIA, MI and death after TAVR in DAPT vs. SAPT patients at 3 months, reported in “The ARTE” study (23). DAPT, dual antiplatelet therapy; SAPT, single antiplatelet therapy; LT, life-threatening; TIA, transient ischemic attack; MI, myocardial infarction.

Antithrombotic strategy is particularly challenging because TAVR patients are usually at high risk of both bleeding and ischemic events. Today, in absence of clear indications for therapeutic anticoagulation, DAPT for 1–6 months followed by SAPT lifelong in patients without an indication for oral anticoagulation (OAC) has been empirically recommended by a consensus of TAVR experts (16). The differences in the duration of antithrombotic therapy and all data about antithrombotic treatment post-TAVR are limited to observational studies and very few RCTs (14, 17, 18). The duration of DAPT varied widely among centers (1, 3, 6, 12 months and indefinitely in 14.2, 41, 32.6, 5, and 1.3% of centers). A minority of centers (6.7%) reported the systematic use of SAPT with aspirin alone. High variability in antithrombotic regimes was observed in patients with AF between centers: warfarin alone, warfarin + clopidogrel, warfarin + aspirin, and triple therapy were used in 27.9, 25.9, 38.9, and 4.5% of the centers, respectively (Figure 2) (19). Several larger randomized studies are currently ongoing and should provide evidence-based data with respect to the optimal antithrombotic therapy strategy after TAVR. To date, a small pilot study suggested no difference regarding thromboembolic and bleeding complications whereas three retrospective studies showed a lower bleeding risk between aspirin alone and DAPT strategy after TAVR (17, 20–22). To date, the most recent RCT is “Aspirin vs. Aspirin + Clopidogrel Following Transcatheter Aortic Valve Implantation” (ARTE) where aspirin (80–100 mg/day) plus clopidogrel (75 mg/day) was compared with aspirin alone. A total of 222 patients were randomized (1:1) the day before the TAVR procedure to receive aspirin or acetylsalicylic acid (80–100 mg/day) plus clopidogrel (75 mg/day) or aspirin or acetylsalicylic acid (80–100 mg/day) alone following the TAVR procedure. The rate of major or life-threatening bleeding events at 3 months was higher in the DAPT group (10.8 vs. 3.6%%; OR: 3.22; 95% CI: 1.01–10.34; *p* = 0.038), whereas there were no differences between groups in the incidence of ischemic stroke or TIA (DAPT 2.7%; SAPT 0.9%; OR: 3.11; 95% CI: 0.32–30.43; *p* = 0.313), MI (DAPT 3.6%; SAPT 0.9%; OR: 4.13; 95% CI: 0.45–37.60; *p* = 0.175) or death (DAPT 6.3%; SAPT 3.6%; OR: 1.78; 95% CI: 0.51–6.27; *p* = 0.370) (Figure 3) (23). In the SAT-TAVI (single antiplatelet therapy for TAVI) study 120 consecutive patients, undergoing TAVR, were randomly assigned to acetylsalicylic acid (ASA) group or DAPT group (aspirin plus clopidogrel 75 mg or plus ticlopidine 500 mg). Vascular complications resulted to be more frequent in the DAPT group at 30 days (DAPT 13.3%; SAPT 5%; *p* ≤ 0.05) and there was no difference between groups in the incidence of ischemic stroke or TIA (DAPT 1.7%; SAPT 1.7%; *p* = ns) (18). D'Ascenzo et al. included all consecutive TAVR patients in The Italian Transcatheter Balloon-Expandable Registry (ITER) (a total of 1,210 patient; 605 for each group, aspirin alone vs. DAPT) to compare all-cause death, cardiovascular death, bleedings, vascular complications, and cerebrovascular accidents. At 30 days, rates of VARC-2 mortality were lower in patients with aspirin alone (DAPT 4.1%; SAPT 1.5%; *p* = 0.003), mainly driven by a reduction of major bleedings (DAPT 11.5%; SAPT 6.6%; *p* < 0.001) and major vascular complications (DAPT 10.7%; SAPT 5.3%; *p* < 0.001) (24).

**Figure 4:**
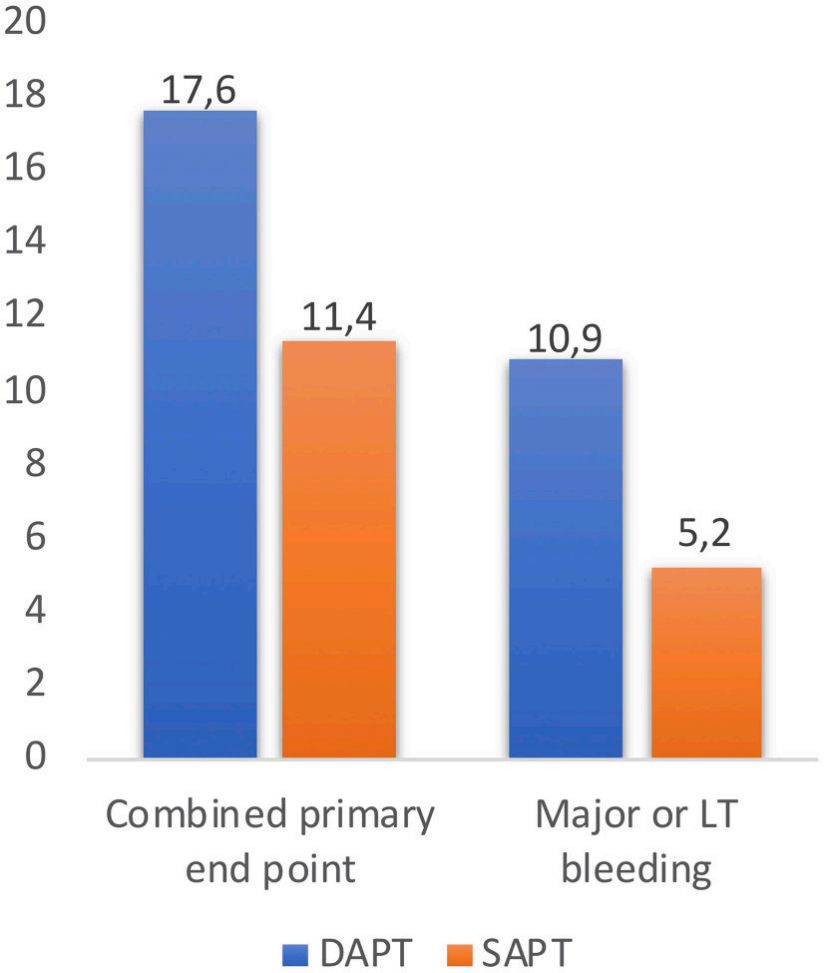
The occurrence of the 30-day combined primary end point (occurrence of death, major, or life-threatening bleedings, and major vascular complications at 30-day follow-up) and rate of major or life-threatening bleeding events after TAVR, reported in the study “Meta-Analysis Comparing Single vs. Dual Antiplatelet Therapy Following Transcatheter Aortic Valve Implantation (25).” DAPT, dual antiplatelet therapy; SAPT, single antiplatelet therapy; LT, life-threatening.

More recently, data from the 3 randomized trials comparing DAPT vs. SAPT in 421 non-OAC patients post-TAVR were pooled and analyzed in a meta-analysis (25). The primary end point was the occurrence of death, major or life-threatening bleedings, and major vascular complications at 30-day follow-up, based on the VARC-2 definitions (8). The occurrence of the 30-day combined primary end point (occurrence of death, major or life-threatening bleedings, and major vascular complications at 30-day follow-up) was higher in the DAPT group (17.6 vs. 10.9%; OR: 1.73; 95% CI: 1.00–2.98, *p* = 0.050), with an increased rate of major or life-threatening bleeding events in the DAPT group (11.4 vs. 5.2%; OR:2.24, 95% CI: 1.12–4.46, *p* = 0.022) (Figure 4) (25). These results may suggest DAPT is related with a higher rate of major adverse events after TAVR, determined by an increased risk of major or life-threatening bleeding complications with a lack of beneficial effect in the incidence of ischemic stroke or TIA, MI and death. Furthermore, it seems that there are no relevant differences between SAPT and DAPT regarding risk of thromboembolism and valve dysfunction at mid-term follow-up (18, 23). Several larger randomized studies are currently ongoing and should provide evidence-based data with respect to the optimal antithrombotic therapy strategy during and after TAVR.

In addition, about one-third of patients undergoing TAVR require an oral anticoagulant, typically for atrial fibrillation (AF). To date, there are two ongoing big trials that compare DAPT with SAPT in patients after TAVR, Antiplatelet Therapy for Patients Undergoing Transcatheter Aortic Valve Implantation (POPular-TAVI) and CLOE. The POPular-TAVI is the first RCT to test both aspirin and OAC therapy with the currently recommended supplement of clopidogrel after TAVR for 3 months. It encompasses 2 cohorts, cohort A with patients without an indication for OAC (randomized aspirin <100 mg/day, minimum 1 year vs. aspirin <100 mg/day, minimum 1 year plus clopidogrel 75 mg, 3 months) and cohort B with an indication for OAC (OAC alone vs. OAC plus clopidogrel 75 mg, 3 months) at the time of randomization (26). The CLOE trial will start in the US to estimate the safety and efficacy of clopidogrel in patient with and without indication for OAC after TAVR. Two cohorts will be randomized, cohort A with patients without an indication for OAC (randomized aspirin alone vs. aspirin plus clopidogrel > 6 months) and cohort B with patients with an indication for OAC (OAC alone vs. OAC plus clopidogrel > 6 months).

Regarding OAC therapy after TAVR in patients with sinus rhythm, it has to be mentioned the early stop of “Global Study Comparing a rivaroxaban-based Antithrombotic Strategy to an antiplatelet-based Strategy After Transcatheter aortic valve replacement to Optimize Clinical Outcomes” (GALILEO) trial due to safety concerns. This study investigated the clinical benefits of a rivaroxaban-based anticoagulation strategy (rivaroxaban 10 mg once daily plus aspirin 75–100 mg once-daily for 3 months followed by rivaroxaban alone), or an antiplatelet strategy (clopidogrel 75 mg plus aspirin 75–100 mg once daily for 3 months followed by aspirin alone) in patients without indication for OAC. The data and safety monitoring board of the GALILEO trial have halted this study because the data showed that rivaroxaban-based anticoagulation strategy was associated with an excess of bleeding, without a proportionate reduction in ischemic events in unadjusted analysis (Table 2) (27). Ongoing trials are expected to draw a clearer picture on the field.

**Table 2:** Main randomized trials evaluating antithrombotic regimen after TAVR.

**Trials**	**Antithrombotic regimen**	**Patients randomized**	**Target patients**	**Status**
Ussia et al.	Aspirin plus clopidogrel vs. Aspirin alone	79	Patients without indication for OAC	Published in December 2011
SAT-TAVI Trial	Aspirin plus clopidogrel or ticlopidine vs. Aspirin alone	120	Patients without indication for OAC	Published in July 2014
The ARTE randomized clinical trial	Aspirin plus clopidogrel vs. Aspirin alone	222	Patients without indication for OAC	Published in July 2017
GALILEO	Rivaroxaban plus Asa for 90 days followed by rivaroxaban alone vs. Clopidogrel plus ASA for 90 days followed by Aspirin alone	~1,520	Patients without indication for OAC	The Trial has been halted on October 2018
POPular-TAVI (NCT02247128)	**Cohort A** Aspirin plus clopidogrel vs. Aspirin alone	1,000	Patients without an indication for OA	Expected publication: March 2020
	**Cohort B** OAC plus clopidogrel vs. OAC alone		Patients with an indication for OAC	
ATLANTIS (NCT02664649)	Apixaban vs. Standard of Care	1,510	All type of patients	Expected publication: May 2020
AVATAR (NCT02735902)	Anticoagulation alone vs. Anticoagulation and Aspirin	170	Patients with indication for OAC	Expected publication: April 2020
ENVISAGE-TAVI AF (NCT02943785)	Edoxaban vs. Standard of Care	1,400	Patients with AF prior to TAVR	Expected publication: November 2020
CLOE	**Cohort A** Aspirin plus clopidogrel vs. Aspirin alone	~4,000	Patients without an indication for OAC	Announced
	**Cohort B** OAC plus clopidogrel vs. OAC alone		Patients with an indication for OAC	

*OAC, oral anticoagulant*.

## Post-Procedural Antithrombotic Therapy for TAVR Patients With Atrial Fibrillation

Patients with atrial fibrillation (AF) undergoing TAVR represent a unique management challenge. In literature, AF was documented in about a third of patients before TAVR (28). An analysis from the STS/ACC TVT registry showed that post-TAVR, new onset of AF occurred in 8.4% of patients (4.4% with TF access, 16.5% with non-TF access) (29). Several studies showed that both pre-existing and new-onset AF in TAVR patients has been associated with higher rates of mortality at 1 year (29, 30). Unfortunately, there are no clear recommendations on the use of long-term antiplatelet treatment on top of anticoagulation therapy in patients with AF undergoing TAVR. The ESC/EACTS 2017 expert consensus suggests, despite the lack of evidence, a combination of vitamin K antagonist (VKA) and aspirin or thienopyridine but it should be weighed against an increased risk of bleeding (31). The ACCF/ AATS/ SCAI/STS 2012 Expert Consensus suggests that in patients treated with warfarin, a direct thrombin inhibitor, or factor Xa inhibitor, it is reasonable to continue low-dose aspirin but other antiplatelet therapy should be avoided if possible (14); finally, the Canadian Cardiovascular Society 2012 Position Statement suggests that the need for adjunctive antiplatelet agents is controversial, and triple therapy should be avoided unless definite indications exist (32). However, the potential risk of bleeding complications may clearly outweigh that of thromboembolism after TAVR when adding antiplatelet treatment in patients with OAC indication. Currently, in the TAVR field there are no studies focusing exclusively on patients with indication to oral anticoagulants (warfarin or non-vitamin K antagonists), but many trials are underway. The Oral anti-Xa anticoagulation after trans-aortic valve implantation for aortic stenosis: The randomized “ATLANTIS trial,” “Anticoagulation Alone vs. Anticoagulation and Aspirin Following Transcatheter Aortic Valve Interventions” (AVATAR), “Edoxaban vs. standard of care and their effects on clinical outcomes in patients having undergone Transcatheter Aortic Valve Implantation in Atrial Fibrillation (ENVISAGE-TAVI AF) trial,” and POPular-TAVI (cohort B) trials (26) will include patients with AF and will add new information regarding the best treatment strategy for this kind of patients. ATLANTIS will test the superiority apixaban-based strategy vs. the recommended standard of care strategy to reduce the risk of thromboembolic and bleeding complications after TAVR (6). AVATAR will try to demonstrate that SAPT is superior to a combination of anticoagulant and antiplatelet therapy on the net clinical benefit estimated at 1 year after the procedure (33). The ENVISAGE-AF trial will compare a traditional VKA-based strategy with edoxaban (60 mg/day) and antiplatelet therapy in approximately 1,400 patients with an indication for OAC after successful transfemoral TAVR (Table 2) (34). These new trials will delineate the best antithrombotic treatment in patients with AF before and after TAVR. For the patients with an increased bleeding risk, the left atrial appendage occlusion may be an alternative to OAC. The best candidates could be those with chronic AF, contraindications to OAC or a high risk of drug–drug interaction, and high bleeding risk, like patients with coronary artery stenting that need concomitant DAPT with the risk of prolonged triple therapy (35, 36). This approach has been demonstrated to be safe in recent small series (35, 36).

## Bioprosthetic Leaflets Thrombosis

**Figure 5:**
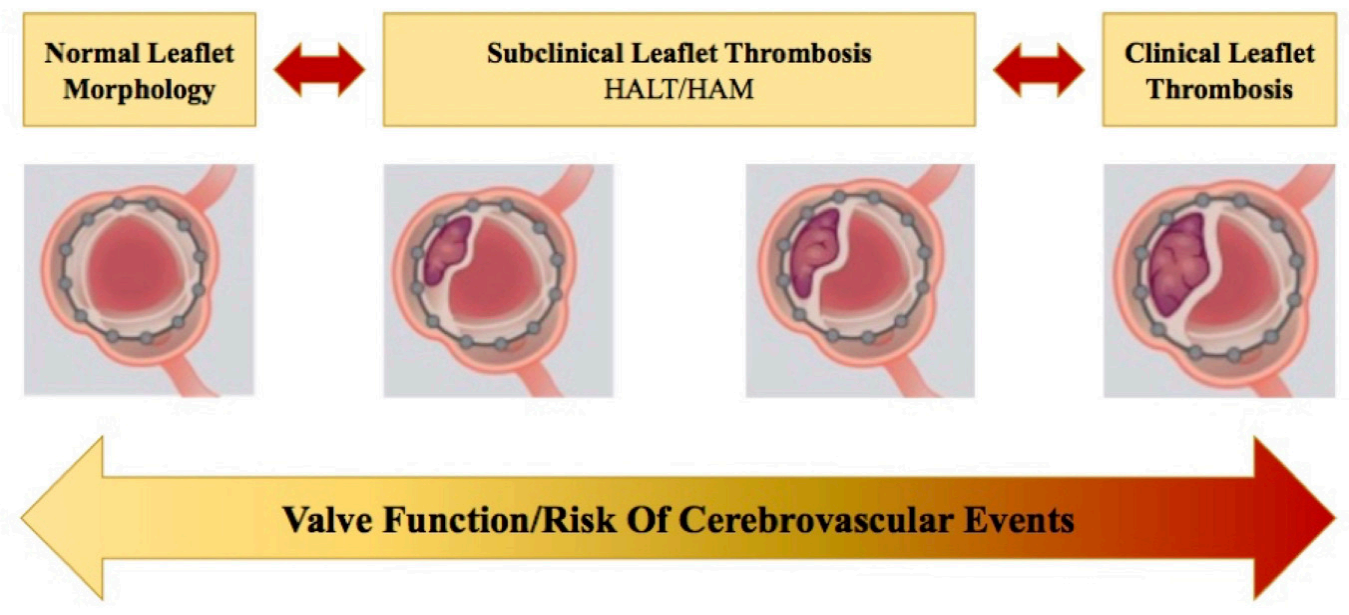
Natural progression and/or regression of subclinical leaflet thrombosis (SLT) in transcatheter heart valves (THV). When left untreated, this may lead to clinically overt leaflet thrombosis, leading to structural valve deterioration and a potential increase in the risk of cerebrovascular events. HALT, hypo attenuated leaflet thickening; HAM, hypoattenuation affecting motion (38).

To date, there is increasing evidence in literature that identifies early thrombus stratification upon transcatheter aortic valve (TAV) leaflets as the first stage of bioprosthesis degeneration process (37). Subclinical leaflet thrombosis (SLT) has the hallmark features of hypo attenuated leaflet thickening (HALT) on multidetector computed tomography (MDCT), which may result in hypoattenuation affecting motion (HAM) (Figure 5) (38–41). The pathophysiological mechanisms underlying SLT are not well**-**understood. The Virchow's triad describes 3 factors in the pathogenesis of thrombosis—surface damage, hemodynamic flow alteration, and hypercoagulable state. Firstly, *in vitro* studies suggested that valve crimping and expansion (in balloon-expandable devices) may lead to irregular leaflet surfaces, micro filamentous damage and reduced integrity of the leaflets (42). Exposed collagen leads to increased surface thrombogenicity and consequently platelet activation (43). Secondly, a low cardiac output state leads to reduced transprosthetic flow, which promotes hypercoagulability by disrupting the balance of activated clotting factors and inhibitors on the leaflet surface (blood stasis leads to a greater increase in clotting factors over inhibitors) (44). Moreover, local flow turbulence and disturbance at the level of the leaflet surface may promote platelet adhesion and activation. Lastly, patients undergoing TAVR may also have comorbidities associated with the development of thromboembolism (advanced age, diabetes, chronic kidney disease, and inflammatory conditions). In literature, data about the best strategy to prevent SLT in TAV remain extremely scarce. A recent analysis of the SAVORY registry exhibited that the use of an anticoagulation therapy post TAVR has been shown to reduce the risk of developing SLT when compared with conventional antiplatelet therapy (40).

## Conclusion

**Figure 6:**
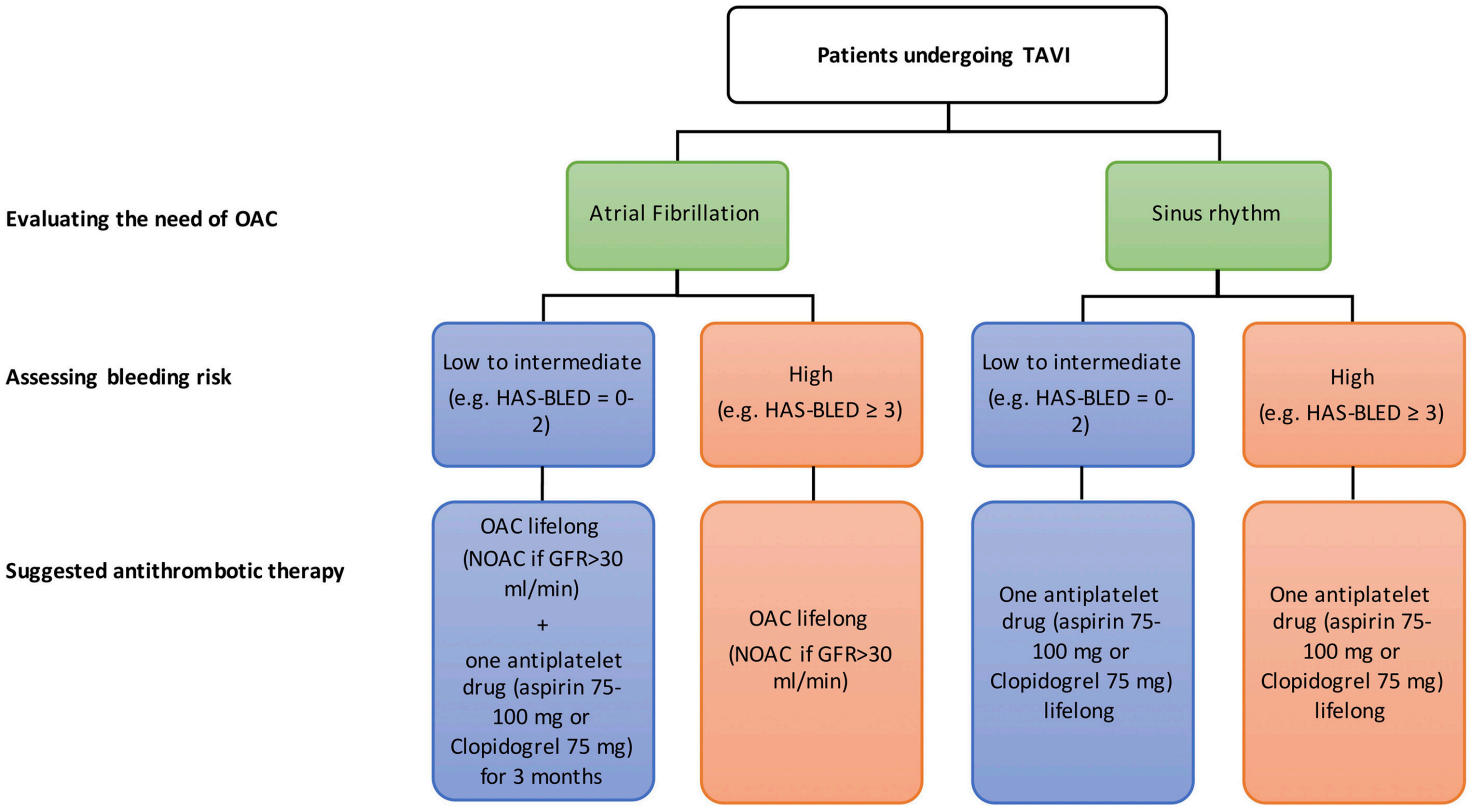
Institutional approach for antiplatelet therapy following TAVR.

Currently, the use of DAPT with clopidogrel for 1–6 months followed by aspirin lifelong is the most popular antithrombotic treatment for all patients without an indication for OAC after TAVR. This strategy is mainly based on experience from coronary and peripheral vascular therapies but evidence of additional protection from ischemic complications are missing. Furthermore, there is a growing amount of evidence for SAPT alone after TAVR as it appears to be safer in terms of bleeding when compared to DAPT. In Figure 6, we depicted institutional approach to antithrombotic therapy after TAVR. Limited data are available regarding the optimal antithrombotic therapy in patients undergoing TAVR with a clear indication to OAC. Use of OAC for reducing TAVR-related thromboembolic risk and bioprosthetic leaflets thrombosis is still debatable and RCTs are needed in this field. Full results from ongoing randomized trials will improve our current limited knowledge on the optimal antithrombotic treatment after TAVR and help to build up dedicated practice guidelines.

## Author Contributions

RV and GC provided the first revision of the manuscript. CT and MB made critical revisions of the text and gave final approval.

### Conflict of Interest Statement

MB is consultant for Edwards Lifesciences and was an advisory board member for Biotronik. CT received speaker honoraria from Medtronic, Boston Scientific, Edwards Lifesciences and Abbott Vascular. The remaining authors declare that the research was conducted in the absence of any commercial or financial relationships that could be construed as a potential conflict of interest.
