# Supplementary material for: Antithrombotic Therapy in Transcatheter Aortic Valve Replacement
Source: Front Cardiovasc Med. 2019 May 31;6:73. doi: 10.3389/fcvm.2019.00073 (PMC6554284; doi:10.3389/fcvm.2019.00073)
Supplement: Supplementary file 1 [file Table_1.DOCX]

Supplementary Material

# Supplementary Tables

Table 1: Definitions of Bleeding and Cerebrovascular Events (VARC 2 Criteria).

| **Complication** | **Definition** |
| --- | --- |
| **Cerebrovascular event** | Acute episode of a focal or global neurological deficit with at least 1 of the following: change in level of consciousness, hemiplegia, hemiparesis, numbness or sensory loss affecting 1 side of the body, dysphasia or aphasia, hemianopia, amaurosis fugax, or other neurological signs or symptoms consistent with stroke. No other readily identifiable non‒stroke cause for the clinical presentation (e.g., brain tumor, trauma, infection, hypoglycemia, peripheral lesion, pharmacological influences), to be determined by or in conjunction with designated neurologist* Confirmation of the diagnosis by at least 1 of the following: neurologist or neurosurgical specialist, or neuroimaging procedure (imaging procedure MRI or CT scan), but stroke may be diagnosed on clinical grounds alone. |
| **Stroke** | Duration of a focal or global neurological deficit ≥24 h; or available neuroimaging documents a new hemorrhage or infarct; OR the neurological deficit results in death A stroke may be classified as undetermined classification, if there is insufficient information to allow categorization as ischemic or hemorrhagic. |
| **Ischemic** | An acute episode of focal cerebral, spinal, or retinal dysfunction caused by infarction of central nervous system tissue. |
| **Hemorrhagic** | An acute episode of focal or global cerebral or spinal dysfunction caused by intraparenchymal, intraventricular, or subarachnoid hemorrhage. |
| **Major (disabling)** | mRS score of ≥2 at 90 days and an increase of ≥1 mRS category from the patient’s pre-stroke baseline. |
| **Minor (non- disabling)** | mRS score of <2 at 90 days, or one that does not result in an increase of ≥1 mRS category from the patient’s pre-stroke baseline. |
| **Transient ischemic attack (TIA)** | Duration of a focal or global neurological deficit <24 h. Any variable neuroimaging does not demonstrate a new hemorrhage or infarct. |
| **Life-threatening or disabling Bleeding** | Fatal bleeding (BARC type 5), OR Bleeding in a critical organ, such as intracranial, intraspinal, intraocular, or pericardial necessitating pericardiocentesis, or intramuscular with compartment syndrome (BARC type 3b and 3c), OR Bleeding causing hypovolemic shock or severe hypotension requiring vasopressors or surgery (BARC type 3b), OR Overt source of bleeding with drop in hemoglobin of ≥5 g/dl or whole blood or packed red blood cell transfusion ≥4 units‡ (BARC type 3b) |
| **Major Bleeding** | (BARC type 3a) Overt bleeding either associated with a drop in the hemoglobin level of ≥3.0 g/dl or requiring transfusion of 2 or 3 units of whole blood/packed red cells, or causing hospitalization or permanent injury, or requiring surgery AND Does not meet criteria of life-threatening or disabling bleeding. |
| **Minor Bleeding** | (BARC type 2 or 3a, depending on the severity) Any bleeding worthy of clinical mention (e.g., access site hematoma) that does not qualify as life-threatening, disabling, or major. |

*Patients with nonfocal global encephalopathy will not be reported as a stroke without unequivocal evidence of cerebral infarction based upon neuroimaging studies (computed tomography [CT] scan or brain magnetic resonance imaging [MRI]). ‡Given that 1 unit of packed red blood cells typically will raise blood hemoglobin concentration by 1 g/dl, an estimated decrease in hemoglobin will be calculated. Abbreviations: BARC, Bleeding Academic Research Consortium; mRS, modified Rankin Scale; VARC, Valve Academic Research Consortium.
